# Serum 25-Hydroxyvitamin D Status and 12-Week Functional Outcomes After Extracorporeal Shock Wave Therapy for Lateral Epicondylitis: A Retrospective Cohort Study

**DOI:** 10.3390/nu18132152

**Published:** 2026-07-02

**Authors:** Ki-Hyeok Ku, Eo Jin Park

**Affiliations:** 1 Department of Orthopedic Surgery, Kyung Hee University College of Medicine, Kyung Hee University Hospital at Gangdong, Seoul 05278, Republic of Korea; 2 Department of Rehabilitation Medicine, Kyung Hee University College of Medicine, Kyung Hee University Hospital at Gangdong, Seoul 05278, Republic of Korea

**Keywords:** lateral epicondylitis, 25-hydroxyvitamin D, vitamin D deficiency, QuickDASH, grip strength, extracorporeal shock wave therapy, ultrasonography

## Abstract

Background/Objectives: Serum 25-hydroxyvitamin D [25(OH)D] is a clinically used biomarker of vitamin D nutritional status, although it is also influenced by sunlight exposure, supplementation, season, and other host factors. Short-term functional status after extracorporeal shock wave therapy (ESWT) for lateral epicondylitis varies. We evaluated whether serum 25(OH)D level and status were associated with 12-week functional outcomes among ESWT-treated patients. Methods: This single-center retrospective cohort included 62 adults with lateral epicondylitis who received outpatient ESWT and had baseline and 12-week assessments. Baseline variables included grip strength ratio, Quick Disabilities of the Arm, Shoulder and Hand (QuickDASH) score, serum 25(OH)D measured using the Architect 25-OH D vitamin kit, common extensor tendon (CET) thickness, age, sex, and body mass index. Multivariable linear regression was used in an analysis-of-covariance framework. Serum 25(OH)D was assessed continuously and as <20 versus ≥20 ng/mL in exploratory threshold analysis. Results: Serum 25(OH)D was 21.0 ± 8.4 ng/mL; 30 patients (48.4%) had <20 ng/mL, 22 (35.5%) had 20–29.9 ng/mL, and 10 (16.1%) had ≥30 ng/mL. QuickDASH decreased from 42.0 ± 17.4 to 27.0 ± 13.7, and grip strength ratio increased from 0.58 ± 0.14 to 0.76 ± 0.14. Higher serum 25(OH)D was associated with lower 12-week QuickDASH after adjustment (β per 10 ng/mL = −4.04, 95% CI −7.17 to −0.91; *p* = 0.012). Additionally, 25(OH)D <20 ng/mL was associated with higher 12-week QuickDASH (β = 6.43, 95% CI 1.17 to 11.69; *p* = 0.017). Serum 25(OH)D was not clearly associated with 12-week grip strength ratio. Conclusions: Lower serum 25(OH)D, interpreted as a vitamin D nutritional-status marker rather than as a nutrition-specific causal exposure, was associated with worse 12-week patient-reported function, but not grip strength ratio. The <20 ng/mL threshold analysis was exploratory and was not powered for subgroup inference. These findings should be interpreted as observational and hypothesis-generating.

## 1. Introduction

Lateral epicondylitis is a common cause of lateral elbow pain and upper-extremity disability in adults, particularly in individuals exposed to repetitive gripping, wrist extension, or forearm loading [1,2]. The problem is typically recognized as a tendinopathic condition, including degenerative changes at the common extensor origin, rather than merely an inflammatory process, despite the continued frequent use of the term “epicondylitis” in clinical practice [3,4]. In addition to showing diminished grip strength and poor upper extremity function, patients frequently experience pain during everyday activities, work-related duties, and sports [1,5]. In routine outpatient care, it is still necessary to better characterize baseline characteristics associated with treatment outcomes because individuals’ clinical presentations and recovery trajectories differ significantly [6,7].

Lateral epicondylitis has been treated nonoperatively using a variety of methods, such as activity adjustment, exercise-based rehabilitation, bracing, oral analgesics, injections, and extracorporeal shock wave therapy (ESWT) [8,9]. ESWT continues to garner clinical interest because it is noninvasive, can be administered in an outpatient setting, and has been utilized as a substitute for patients with persistent symptoms [10]. Although some individuals with lateral epicondylitis may benefit from ESWT in terms of pain and function, the short-term results following treatment are not always consistent [10,11,12]. In clinical practice, following ESWT, individuals with seemingly comparable conditions frequently exhibit inconsistent short-term results [11,12]. This diversity implies that baseline clinical status, structural tendon features, and host-related variables may influence follow-up results following ESWT [13,14].

Complementary data on this heterogeneity can be obtained from several baseline measures readily available in outpatient practice. Calculated from the affected and unaffected sides, the grip strength ratio can partially account for inter-individual variations in absolute strength while also reflecting objective functional impairment [15,16]. Elbow diseases are frequently assessed using the Quick Disabilities of the Arm, Shoulder, and Hand (QuickDASH) questionnaire, which provides a patient-reported measure of upper-extremity disability [17,18]. Additionally, common extensor tendon (CET) thickening is frequently seen in lateral epicondylitis, and musculoskeletal ultrasonography is a useful tool for assessing tendon anatomy [19,20]. However, the extent to which sonographic CET thickness is associated with short-term prognosis following ESWT remains uncertain, and structural anomalies do not always correlate with symptom severity or functional limitation [20,21,22]. Serum 25-hydroxyvitamin D level may represent an additional nutrition-associated host factor of interest [23]. We selected 25(OH)D because it is the major circulating vitamin D metabolite and the clinically preferred biomarker for assessing vitamin D status, reflecting vitamin D input from diet, supplements, and cutaneous synthesis while having a longer circulating half-life than active 1,25-dihydroxyvitamin D [24]. Vitamin D has been implicated in musculoskeletal health and has also been explored for a potential role in tendon homeostasis and tissue healing [23,25].

When combined, serum 25-hydroxyvitamin D levels, sonographic CET thickness, QuickDASH score, and baseline grip strength ratio may provide additional baseline data relevant to short-term follow-up outcome variations after treatment. They have not, however, been thoroughly studied in relation to short-term functional results following outpatient ESWT for lateral epicondylitis [15,21,22,23]. The aim of this single-center retrospective cohort study was to evaluate whether baseline grip strength ratio, QuickDASH score, serum 25-hydroxyvitamin D level, and common extensor tendon thickness were associated with 12-week QuickDASH score and 12-week grip strength ratio among patients with lateral epicondylitis treated with outpatient ESWT. We postulated that worse 12-week QuickDASH scores and lower 12-week grip strength ratios would be associated with lower baseline grip strength ratios, higher baseline QuickDASH scores, lower serum 25-hydroxyvitamin D levels, and higher CET thicknesses.

## 2. Materials and Methods

### 2.1. Study Design and Participants

This study was designed as a single-center retrospective cohort study based on outpatient records from the departments of Orthopedic Surgery and Physical and Rehabilitation Medicine at Kyung Hee University Hospital at Gangdong. Adults with clinically confirmed lateral epicondylitis who started a standardized outpatient extracorporeal shock wave therapy (ESWT) program and had access to baseline and follow-up functional evaluations were included in the study. The study period was from March 2021 to November 2025. The target follow-up time point was 12 weeks after baseline. To accommodate routine outpatient scheduling in this retrospective cohort, the acceptable follow-up window for this analysis was defined as 84 ± 21 days. Baseline was defined as the assessment completed before initiation of ESWT. This study retrospectively evaluated outcomes among patients who received ESWT as part of routine clinical care rather than through prospective assignment by the investigators, and the findings were therefore interpreted within an observational cohort framework. Patients with a clinical diagnosis of lateral epicondylitis who began outpatient ESWT during the study period and were at least 19 years old were eligible to participate. The baseline Quick Disabilities of the Arm, Shoulder, and Hand (QuickDASH) score, baseline grip strength measurements on both the affected and unaffected sides, serum 25-hydroxyvitamin D measurement obtained within one month of baseline, sonographic measurement of common extensor tendon (CET) thickness obtained within one month of baseline, and both QuickDASH and grip strength measurements at the 12-week follow-up visit were all necessary for patients to be included in the analytic cohort.

Patients were excluded if their bilateral lateral epicondylitis prevented them from calculating the grip strength ratio; if they had previously undergone elbow surgery or had experienced a fracture or dislocation involving the affected elbow; if they received additional invasive treatment during the 0- to 12-week follow-up period, such as corticosteroid injection, platelet-rich plasma injection, or surgery; or if they had a major condition that was expected to significantly affect upper-extremity function, such as severe cervical radiculopathy, peripheral neuropathy, or rheumatologic disease. Additionally, we excluded patients with missing data on important characteristics, such as baseline or 12-week QuickDASH score, baseline or 12-week grip strength, serum vitamin D level, or CET thickness. The final analysis was performed as a complete-case analysis because the study was based on a retrospective review of existing clinical data, and the eligibility criteria required complete information on the main exposures and outcomes. This study included 62 patients who met eligibility criteria. The study protocol was reviewed and approved by the Institutional Review Board of Kyung Hee University Hospital at Gangdong (IRB No. 2026-03-048; approval date, 23 April 2026), and the requirement for informed consent was waived due to the retrospective nature of the study and use of de-identified data.

### 2.2. Data Collection and Measurement Procedures

Clinical measurements were obtained during routine outpatient care by trained clinical staff in the relevant outpatient departments, and the analytic dataset was abstracted retrospectively from electronic medical records by the investigators. Because this was not a prospective measurement protocol, a single dedicated research staff member did not perform all patient-facing assessments. Electronic medical records were used to gather clinical, laboratory, imaging, and follow-up data. Age (years), sex, body mass index (BMI; kg/m^2^), affected side (left or right), dominant arm involvement (yes or no), and symptom duration (months) were collected as baseline clinical and demographic variables.

The clinical diagnosis of lateral epicondylitis was based on lateral elbow pain, tenderness over the lateral epicondyle, and pain provoked by gripping or resisted wrist extension [24,26].

Grip strength was measured in kilograms using a digital hand dynamometer (Grip-D, T.K.K. 5401; SANKA Co., Ltd., Niigata, Japan). As per conventional clinical practice, measurements were obtained with the person seated, elbow flexed to 90°, wrist slightly extended, shoulder adducted, and forearm in a neutral position [27,28]. Each side underwent three trials, and the mean value was used for analysis [27,29]. The grip strength ratio, calculated by dividing the affected-side grip strength by the unaffected-side grip strength, was calculated using baseline grip strength measurements [30,31]. This ratio was utilized to partially account for inter-individual variance in absolute strength while reflecting side-to-side functional impairment [30,31].

QuickDASH was used as a patient-reported indicator of upper-extremity disability. Baseline QuickDASH scores ranged from 0 to 100, with higher scores denoting severe disability [17,32]. QuickDASH scores were calculated according to the standard scoring method and recorded as whole numbers after rounding to the nearest integer.

Serum 25(OH)D was measured using the Architect 25-OH D vitamin kit (Abbott Diagnostics, Lake Forest, IL, USA) as part of routine clinical laboratory testing. Serum 25(OH)D was interpreted as a vitamin D nutritional-status biomarker rather than as a direct measure of dietary intake alone. For descriptive analyses, serum 25(OH)D status was categorized as <20 ng/mL, 20–29.9 ng/mL, and ≥30 ng/mL [33]. The <20 ng/mL cut-off was used because it is commonly used to denote vitamin D deficiency in clinical endocrine guidance, whereas the 20–29.9 ng/mL and ≥30 ng/mL categories were retained as descriptive categories rather than treatment targets [33]. Vitamin D supplementation status, dietary vitamin D intake, sunlight exposure, and season of blood sampling were not consistently available in the electronic medical records.

The physician used a Philips HD11 system (Koninklijke Philips Electronics N.V., Amsterdam, Netherlands) to conduct the ultrasound assessment. CET thickness was determined using a standardized musculoskeletal ultrasound approach, with the transducer placed longitudinally above the common extensor tendon origin at the lateral epicondyle [19,34]. Baseline CET thickness values documented in medical records were used for analysis.

All patients underwent outpatient ESWT without local anesthesia using a Dornier AR2 electromagnetic-type ESWT system (Dornier MedTech GmbH, Weßling, Germany) [35]. Shock waves were applied over the lateral epicondyle at the common extensor tendon origin, and the applicator was positioned at the point of maximal tenderness [24,36]. Each treatment session consisted of 2000 shocks with an energy flux density of 0.10–0.15 mJ/mm^2^, administered once weekly for four sessions [37,38]. Other device-adjustable parameters, including pulse frequency and coupling pressure, were not consistently documented in the retrospective treatment records and therefore could not be analyzed separately. Braces and routine oral analgesics were not part of the standard institutional treatment protocol during the follow-up period, although additional self-management measures or treatments received outside the study setting may not have been fully captured in this retrospective review. During the 12-week follow-up phase, the patients were instructed to refrain from further invasive treatments and were treated in accordance with standard outpatient procedures.

### 2.3. Outcome Measures

The QuickDASH score at 12 weeks post-baseline was the primary outcome. Because patient-reported upper-extremity function was considered the most clinically relevant short-term endpoint after outpatient ESWT, this outcome was selected. A higher QuickDASH score at 12 weeks indicated a worse functional result, as QuickDASH values range from 0 to 100, with higher scores indicating greater disability [17]. Although lateral elbow pain is clinically important in lateral epicondylitis, a separate pain-specific instrument, such as a visual analog scale or numeric rating scale, was not consistently recorded in the retrospective medical records. Therefore, pain intensity could not be analyzed as a separate outcome or covariate.

The grip strength ratio at 12 weeks, calculated by dividing the grip strength of the affected side by that of the unaffected side, was the secondary outcome [30]. In addition to the patient-reported primary outcome, this outcome was selected as an objective functional assessment. Compared with the contralateral side, a lower grip strength ratio after 12 weeks suggests poorer recovery of grip function [15,30].

QuickDASH score change and grip strength ratio change from baseline to 12 weeks were calculated for descriptive purposes. QuickDASH change was defined as the 12-week QuickDASH score minus the baseline QuickDASH score, with more negative values indicating greater improvement. Grip strength ratio change was defined as the 12-week grip strength ratio minus the baseline grip strength ratio, with more positive values indicating greater improvement. To aid clinical interpretation, the proportion of patients achieving published QuickDASH minimal important difference or minimal clinically important difference thresholds was also summarized descriptively. Published estimates include approximately 7–9 points in patients with tennis elbow and approximately 12–15 points in broader upper-extremity musculoskeletal populations [39,40]. Because the main analytic approach used analysis of covariance with adjustment for the corresponding baseline value, these change variables were not used as dependent variables in the main regression models.

### 2.4. Exposure/Predictor Measures

Baseline grip strength ratio, QuickDASH score, serum 25(OH)D level, and CET thickness were the baseline variables of major interest. These variables were chosen because they represent clinically available functional, patient-reported, laboratory, and sonographic measurements that may help characterize heterogeneity in short-term follow-up status among patients treated with outpatient ESWT. In the QuickDASH outcome model and grip strength ratio outcome model, baseline grip strength ratio, baseline QuickDASH score, serum 25(OH)D level, and CET thickness were entered as continuous variables. To improve interpretability, regression coefficients are presented per 0.1 increase in baseline grip strength ratio, per 10-point increase in baseline QuickDASH, and per 10 ng/mL increase in serum 25(OH)D. Serum 25(OH)D was also evaluated in exploratory threshold analyses using <20 ng/mL versus ≥20 ng/mL, and in an additional three-category analysis using <20 ng/mL, 20–29.9 ng/mL, and ≥30 ng/mL. To evaluate the robustness of the QuickDASH and grip strength ratio outcome findings after further adjustment for chronicity, symptom duration was included in a supportive sensitivity analysis. Dominant arm involvement was also included in an additional supportive sensitivity analysis. These variables were not included in the main outcome models because of the modest sample size and the aim to avoid overfitting.

### 2.5. Statistical Analysis

R (version 4.2.3; R Foundation for Statistical Computing, Vienna, Austria) was used for all statistical analyses. Because this was a retrospective cohort based on available eligible patients, no a priori sample size calculation was performed. To address sample size adequacy, a post hoc power sensitivity calculation was performed using G*Power 3.1 settings for linear multiple regression with a fixed-model R^2^ increase test. With 62 participants, α = 0.05, 80% power, one tested predictor, and seven total predictors, the minimum detectable effect size was approximately f^2^ = 0.13 for a single tested predictor. For the overall seven-predictor model, 62 participants provided 80% power to detect an effect size of approximately f^2^ = 0.263. Therefore, the study was considered adequately powered only for moderate-to-large regression effects, and all subgroup, threshold, and sex-specific analyses were interpreted as exploratory. Statistical significance was defined as a two-sided *p*-value < 0.05. Categorical variables are described as counts and percentages, and continuous variables are summarized as mean ± standard deviation. Paired *t*-tests were used descriptively to compare baseline and 12-week QuickDASH scores and grip strength ratios within the analyzed cohort. These paired comparisons were interpreted as descriptive pre-to-post changes rather than controlled estimates of treatment efficacy.

To describe model explanatory performance, both model R^2^ and adjusted R^2^ values were reported. Leave-one-predictor-out incremental R^2^ values were also calculated for each covariate by subtracting the R^2^ of the reduced model without that predictor from the R^2^ of the full model. These values were used descriptively to show the unique contribution of each predictor to the full model and were not used as formal hypothesis tests.

The QuickDASH outcome analysis used multivariable linear regression in an analysis-of-covariance framework, with the 12-week QuickDASH score as the dependent variable and baseline grip strength ratio, baseline QuickDASH score, serum 25(OH)D level, CET thickness, age, sex, and BMI as covariates. This approach estimated associations between baseline variables and 12-week patient-reported function while adjusting for baseline status. The grip strength ratio outcome analysis used multivariable linear regression in an analysis-of-covariance framework, with the 12-week grip strength ratio as the dependent variable and the same covariates as the QuickDASH outcome analysis. This model evaluated associations with the objective functional outcome while adjusting for baseline grip strength ratio.

Regression coefficients, 95% confidence intervals (CIs), *p*-values, model R^2^ values, and adjusted R^2^ values are presented for the multivariable models. Serum 25(OH)D was evaluated as a continuous variable in the QuickDASH outcome model and grip strength ratio outcome model. Exploratory threshold analyses evaluated 25(OH)D deficiency defined as <20 ng/mL versus ≥20 ng/mL. Additional supportive analyses included symptom duration, dominant arm involvement, and three-category serum 25(OH)D status. Sex was retained as a covariate and was further explored in sex-stratified and sex-by-baseline grip strength ratio interaction analyses because sex may be related to both vitamin D-related and musculoskeletal physiology through differences in sex-related hormonal milieu, adiposity/body composition, and muscle strength or functional performance. These analyses were considered exploratory because the study was not powered for definitive subgroup inference.

Normal Q-Q plots and residual-versus-fitted plots were used to evaluate model assumptions. Cook’s distance was used to assess influential observations, the Breusch-Pagan test to assess heteroscedasticity, and variance inflation factors to assess multicollinearity. Model stability was further examined using 5000 non-parametric bootstrap resamples and leave-one-out refitting of the QuickDASH and grip strength ratio outcome models. Because patients with missing values for key research variables were excluded before the final analytic cohort was established, no imputation was performed.

## 3. Results

**Figure 1:**
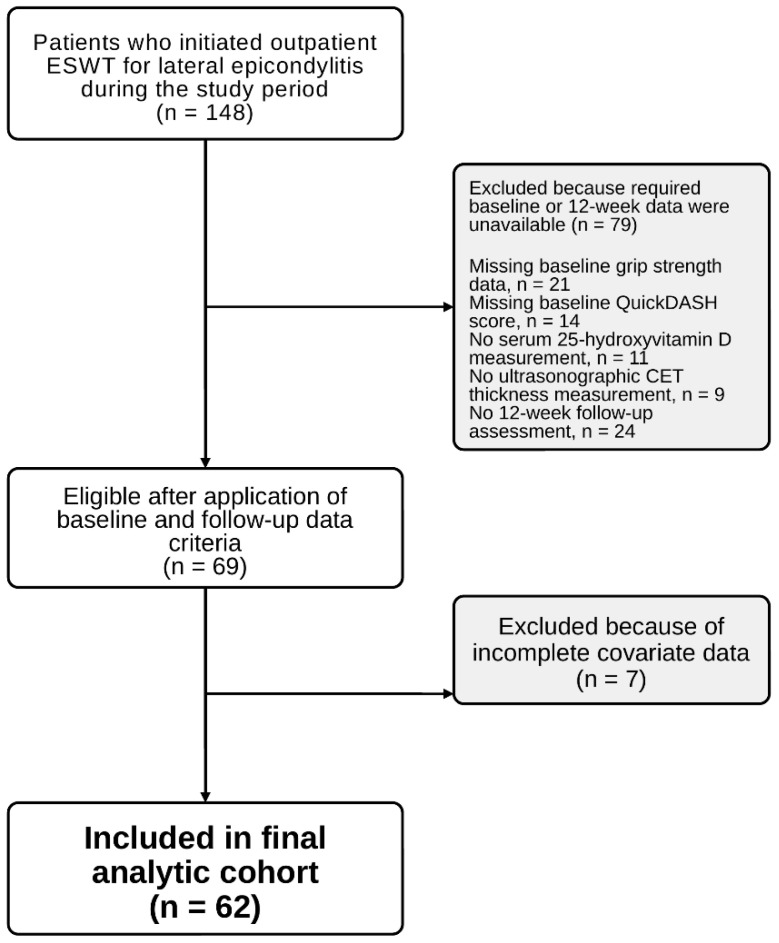
Participant flow diagram. A total of 148 patients who initiated outpatient extracorporeal shock wave therapy (ESWT) for lateral epicondylitis during the study period were screened. Abbreviations: CET, common extensor tendon; ESWT, extracorporeal shock wave therapy; 25(OH)D, 25-hydroxyvitamin D; QuickDASH, Quick Disabilities of the Arm, Shoulder and Hand.

During the study period, 148 patients initiated outpatient ESWT for lateral epicondylitis. The participant flow is shown in Figure 1. Of these, 21 patients were excluded because baseline grip strength data were unavailable, 14 because baseline QuickDASH scores were unavailable, 11 because serum 25(OH)D levels were not measured, 9 because ultrasonographic CET thickness data were unavailable, and 24 because 12-week follow-up assessments were not available. After these exclusions, 69 patients remained. Seven patients were then excluded after application of the clinical exclusion criteria or because complete covariate information was unavailable. The final analytic cohort included 62 patients (Table 1).

The functional outcomes at baseline and 12 weeks are shown in Table 2. The mean QuickDASH score decreased from 42.0 ± 17.4 at baseline to 27.0 ± 13.7 at 12 weeks, corresponding to a mean paired change of −15.0 points (95% CI, −19.7 to −10.3; paired *t*-test *p* < 0.001). Using published QuickDASH interpretability thresholds, 43 patients (69.4%) achieved at least a 7-point improvement, 38 patients (61.3%) achieved at least a 9-point improvement, 35 patients (56.5%) achieved at least a 12-point improvement, and 32 patients (51.6%) achieved at least a 15-point improvement. The mean grip strength ratio increased from 0.58 ± 0.14 at baseline to 0.76 ± 0.14 at 12 weeks, with a mean paired change of 0.17 (95% CI, 0.15 to 0.20; paired *t*-test *p* < 0.001). These descriptive changes indicate lower patient-reported disability and higher side-to-side grip strength ratio at 12 weeks, but they should not be interpreted as a controlled estimate of treatment efficacy.

A multivariable linear regression analysis for the QuickDASH outcome examined baseline variables associated with the QuickDASH score at 12 weeks (Table 3). After adjustment, a 0.1 higher baseline grip strength ratio was associated with a 3.91-point lower QuickDASH score at 12 weeks (β = −3.91, 95% CI −5.88 to −1.95; *p* < 0.001). A 10-point higher baseline QuickDASH score was associated with a 3.80-point higher 12-week QuickDASH score (β = 3.80, 95% CI 2.19 to 5.41; *p* < 0.001). A 10 ng/mL higher serum 25(OH)D level was associated with a 4.04-point lower 12-week QuickDASH score (β = −4.04, 95% CI −7.17 to −0.91; *p* = 0.012). Greater CET thickness was also associated with higher 12-week QuickDASH (β = 5.25 per 1 mm, 95% CI 3.05 to 7.46; *p* < 0.001). Age, sex, and BMI were not clearly associated with 12-week QuickDASH. The full model R^2^ was 0.527 and the adjusted R^2^ was 0.465. Leave-one-predictor-out incremental R^2^ values were 0.139 for baseline grip strength ratio, 0.196 for baseline QuickDASH, 0.059 for serum 25(OH)D, 0.200 for CET thickness, 0.002 for age, 0.009 for sex, and <0.001 for BMI.

A multivariable linear regression analysis for the grip strength ratio outcome evaluated baseline variables associated with the grip strength ratio at 12 weeks (Table 4). A 0.1 higher baseline grip strength ratio was associated with a 0.070 higher 12-week grip strength ratio (95% CI 0.052 to 0.087; *p* < 0.001). A 10-point higher baseline QuickDASH score was associated with a 0.019 lower 12-week grip strength ratio (95% CI −0.033 to −0.004; *p* = 0.011). Serum 25(OH)D showed a positive but non-significant association with the 12-week grip strength ratio (β per 10 ng/mL = 0.023, 95% CI −0.005 to 0.050; *p* = 0.110). CET thickness also did not reach statistical significance in this model (β = −0.019, 95% CI −0.038 to 0.001; *p* = 0.060). The full model R^2^ was 0.618 and the adjusted R^2^ was 0.568. Leave-one-predictor-out incremental R^2^ values were 0.452 for baseline grip strength ratio, 0.049 for baseline QuickDASH, 0.019 for serum 25(OH)D, 0.026 for CET thickness, 0.002 for age, 0.009 for sex, and 0.015 for BMI.

Exploratory threshold analyses evaluated whether serum 25(OH)D deficiency was associated with 12-week outcomes (Table 5). Compared with patients with serum 25(OH)D ≥20 ng/mL, those with serum 25(OH)D <20 ng/mL had a 6.43-point higher QuickDASH score at 12 weeks after adjustment (95% CI 1.17 to 11.69; *p* = 0.017). In contrast, serum 25(OH)D <20 ng/mL was not clearly associated with 12-week grip strength ratio (β = −0.008, 95% CI −0.056 to 0.039; *p* = 0.723). Because the threshold analysis was exploratory and not adjusted for multiplicity, it was interpreted as supportive rather than confirmatory.

**Figure 2:**
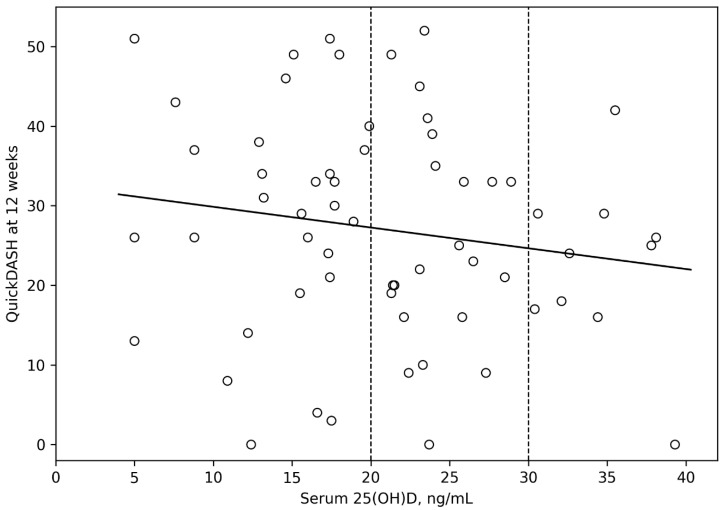
Serum 25(OH)D level and QuickDASH at 12 weeks. The scatterplot shows the unadjusted descriptive relationship between baseline serum 25(OH)D and 12-week QuickDASH. The fitted line represents the unadjusted linear trend, and vertical dashed reference lines indicate 20 and 30 ng/mL. The figure does not display the covariate-adjusted association; adjusted estimates are presented in Table 3, Table 5 and Table 6.

The three-category serum 25(OH)D analysis is shown in Table 6. Compared with the ≥30 ng/mL group, the <20 ng/mL group had a higher 12-week QuickDASH score after adjustment (β = 8.29, 95% CI 0.81 to 15.77; *p* = 0.030). The 20–29.9 ng/mL group did not differ clearly from the ≥30 ng/mL group for 12-week QuickDASH (β = 2.77, 95% CI −5.10 to 10.63; *p* = 0.483). For the 12-week grip strength ratio, neither the <20 ng/mL group nor the 20–29.9 ng/mL group differed clearly from the ≥30 ng/mL group. Because the ≥30 ng/mL group included only 10 patients, the three-category analysis was interpreted as exploratory (Figure 2).

Supportive sensitivity analyses are presented in the Supplementary Materials. After additional adjustment for symptom duration, the direction and magnitude of the main associations were materially unchanged (Supplementary Table S1). Serum 25(OH)D remained associated with 12-week QuickDASH (β per 10 ng/mL = −4.09, 95% CI −7.31 to −0.86; *p* = 0.014), whereas its association with 12-week grip strength ratio remained non-significant (β per 10 ng/mL = 0.021, 95% CI −0.007 to 0.050; *p* = 0.144).

An additional model including dominant arm involvement yielded similar findings, and dominant arm involvement was not associated with either 12-week outcome (Supplementary Table S2).

Exploratory sex-stratified analyses are presented in Supplementary Tables S3 and S4. In sex-stratified models, the association between baseline grip strength ratio and 12-week QuickDASH was directionally similar in females (β per 0.1 increase = −4.27; 95% CI, −6.78 to −1.76; *p* = 0.002) and males (β per 0.1 increase = −3.54; 95% CI, −7.72 to 0.64; *p* = 0.093), although the male subgroup estimate was less precise. Baseline grip strength ratio was also associated with 12-week grip strength ratio in both females (β per 0.1 increase = 0.064; 95% CI, 0.039 to 0.090; *p* < 0.001) and males (β per 0.1 increase = 0.067; 95% CI, 0.034 to 0.100; *p* < 0.001). Sex-by-baseline grip strength ratio interaction terms did not suggest statistically clear effect modification for 12-week QuickDASH (interaction β = 0.94; 95% CI, −3.13 to 5.00; *p* = 0.646) or 12-week grip strength ratio (interaction β = 0.009; 95% CI, −0.027 to 0.045; *p* = 0.620).

For both the QuickDASH and grip strength ratio outcome models, regression diagnostics were evaluated. Variance inflation factors were low, with a maximum value of 1.19, suggesting no major multicollinearity. Visual inspection of normal Q-Q plots and residual-versus-fitted plots did not suggest major deviations from model assumptions. Cook’s distance did not identify observations requiring exclusion, and the Breusch–Pagan test did not suggest substantial heteroscedasticity for the QuickDASH outcome model (*p* = 0.258) or grip strength ratio outcome model (*p* = 0.805). In the 5000-resample bootstrap analysis, the 95% CI for the serum 25(OH)D coefficient in the QuickDASH outcome model was −7.25 to −1.24 per 10 ng/mL. For the grip strength ratio outcome model, the bootstrap 95% CI for the serum 25(OH)D coefficient was −0.007 to 0.050. Leave-one-out refitting did not change the direction of the main QuickDASH associations. Detailed regression diagnostic and bootstrap stability results are provided in Supplementary Table S5.

## 4. Discussion

In this retrospective single-center cohort of patients with lateral epicondylitis treated with outpatient ESWT, baseline grip strength ratio and baseline QuickDASH score showed the most consistent associations with 12-week functional outcomes after adjustment. Lower serum 25-hydroxyvitamin D level and greater CET thickness were associated with worse 12-week QuickDASH, but they were not clearly associated with 12-week grip strength ratio. A higher baseline grip strength ratio was associated with a lower 12-week QuickDASH score and a higher 12-week grip strength ratio, whereas a higher baseline QuickDASH score was associated with a higher 12-week QuickDASH score and a lower 12-week grip strength ratio. Additional adjustment for symptom duration and dominant arm involvement did not materially alter the overall pattern of findings. Taken together, these findings suggest that baseline functional status, and selected biologic or structural characteristics, may help characterize short-term outcome heterogeneity after ESWT for lateral epicondylitis [7,13].

Grip strength ratio and QuickDASH score appeared to be the most consistently informative of the assessed baseline characteristics. The 12-week QuickDASH and 12-week grip strength ratios were both associated with the baseline grip strength ratio after adjustment. The baseline QuickDASH score was likewise associated with both outcomes after adjustment. Because both variables likely capture complementary aspects of the initial disease load, this pattern is clinically plausible [17,41]. While QuickDASH measures the patient’s subjective impact of symptoms on daily upper-extremity function, the grip strength ratio measures objective side-to-side functional impairment and may partially adjust for interindividual differences in absolute strength [41,42,43]. Practically, even after controlling for baseline status and other variables, patients who started therapy with a lower grip strength ratio and higher self-reported impairment had higher residual impairment at 12 weeks after adjustment. These results support the idea that baseline functional severity remains a relevant contextual factor when evaluating short-term functional outcomes following outpatient ESWT rather than suggesting deterministic prediction at the individual level [7,15].

Serum 25-hydroxyvitamin D level and common extensor tendon thickness were also associated with 12-week patient-reported function in the QuickDASH outcome model. Greater tendon thickness was associated with higher QuickDASH scores at follow-up, whereas lower serum 25(OH)D level was associated with worse 12-week QuickDASH scores. These findings suggest that variability in 12-week patient-reported function may not be explained solely by baseline symptom burden, although the absence of corresponding statistically significant associations with grip strength ratio argues for cautious interpretation [7,44]. Common extensor tendon thickness may be interpreted as a pragmatic sonographic structural correlate rather than a comprehensive marker of tendon pathology, whereas serum vitamin D may reflect a broader host-related musculoskeletal state that is not fully captured by questionnaire scores or grip testing alone [14,25,45]. However, in the grip strength ratio outcome model, neither variable reached statistical significance. This outcome-specific pattern may indicate that these baseline factors are more closely reflected in the patient’s reported experience of recovery than in short-term objective grip restoration, or that the current sample size was too small to detect smaller associations with the grip strength ratio outcome [42,46].

From a nutrition-focused perspective, serum 25(OH)D can be interpreted as a clinically accessible marker of vitamin D nutritional status rather than a direct measure of diet alone. It integrates vitamin D from dietary intake, supplement use, and cutaneous synthesis, and is also affected by season, sunlight exposure, adiposity, and other host factors [33]. The present data therefore do not show that nutritional intervention or vitamin D supplementation improves ESWT response. Instead, they suggest that lower vitamin D status may mark a less favorable host musculoskeletal context for patient-reported recovery after ESWT.

It is important to interpret the outcome-specific pattern carefully. Although the grip strength ratio is a more focused performance-based measure, the QuickDASH is a multimodal patient-reported measure that represents pain-related function, activity limitation, and symptom load in everyday life [42,43]. This pattern is clinically plausible because tendon thickness and serum vitamin D level showed clearer associations with QuickDASH than with grip strength ratio. While grip recovery may be more directly dependent on baseline motor performance and residual mechanical capability, patient-reported impairment may be influenced by a wider range of factors, including pain sensitivity, tissue irritability, confidence in limb usage, and perceived functional constraint [44,47,48]. This difference could help explain why vitamin D and common extensor tendon thickness showed only directional but non-significant relationships with the grip strength ratio outcome, whereas the baseline grip strength ratio and QuickDASH continued to be clearly associated. Although the negative coefficient of the grip strength ratio outcome model for common extensor tendon thickness may still be clinically relevant, it would be more accurate to characterize this finding as suggestive rather than definite, given the current dataset.

These relationships may be interpreted through several mechanisms. Reduced voluntary force production, more local pain inhibition, or more severe functional deconditioning of the afflicted limb may contribute to or reflect a lower baseline grip strength ratio [5,41]. A higher baseline QuickDASH score may reflect a greater initial burden of pain-related impairment, which may persist over short-term follow-up [15,32,49]. Increased thickness of the common extensor tendon may indicate structural tendon changes at the tendon origin, possibly indicating altered tendon matrix organization, persistent tendinopathic remodeling, or localized swelling associated with degenerative changes [19,50]. A less favorable musculoskeletal environment, such as impaired tendon homeostasis, muscular performance, or follow-up functional status, may be associated with lower serum 25(OH)D levels [25,51]. Taken together, these observations are compatible with the interpretation that baseline functional, structural, and physiologic domains may mark differences in short-term follow-up status, but they do not establish the mechanisms responsible for recovery patterns.

Sex may also be relevant to the physiologic interpretation of these outcomes. Prior studies suggest that serum 25(OH)D status can differ according to sex and BMI class and that vitamin D status may be associated with sex hormone profiles, including estrogen-related measures, in men and women [52,53]. In addition, sex-associated differences in estrogen-related hormonal milieu, adiposity/body composition, and muscle performance may influence musculoskeletal function and tendon-related recovery. Therefore, sex was retained as an adjustment variable and was also explored in sex-stratified analyses. However, these analyses were exploratory, and the present study cannot determine sex-specific mechanisms.

From a therapeutic standpoint, rather than creating a definitive predictive tool, the current findings may inform pretreatment assessments. The baseline grip strength ratio and QuickDASH score are easy to measure in routine outpatient care and may be useful as baseline descriptors when discussing short-term follow-up status [32,41,49]. Additional context may be provided by serum vitamin D levels and sonographic common extensor tendon thickness, particularly when clinicians consider why patients with seemingly comparable conditions do not recover in the same manner [14,25]. Crucially, available data do not support the creation of risk categories, thresholds, or guidelines for therapy selection. Rather, a more modest interpretation is supported by the results: variation in short-term follow-up status may be characterized by a combination of easily accessible baseline measurements.

The clinical significance of these findings should be interpreted cautiously. The mean QuickDASH improvement of 15.0 points was within or above published minimal important difference ranges for QuickDASH, and approximately one-half to two-thirds of patients achieved commonly used improvement thresholds [39,40]. However, the adjusted vitamin D-related differences were smaller than these patient-level thresholds. For example, the adjusted difference associated with 25(OH)D <20 ng/mL was 6.43 QuickDASH points, and the continuous 25(OH)D coefficient was −4.04 points per 10 ng/mL. Therefore, although the vitamin D associations were statistically significant for 12-week QuickDASH, they should not be interpreted as definitive evidence of clinically meaningful individual-level benefit or as a basis for treatment selection.

This study has several strengths. First, both patient-reported and objective functional outcomes were assessed, allowing the findings for QuickDASH and grip strength ratio to be compared directly. Second, the analysis included clinical, laboratory, and sonographic baseline data that are available in routine outpatient practice. Third, the regression models used an analysis-of-covariance framework with adjustment for the corresponding baseline outcome value. Fourth, the main findings were generally consistent after additional adjustment for symptom duration and dominant arm involvement.

This study has several limitations. Because clinical measurements were obtained during routine outpatient care by trained clinical staff rather than by a single dedicated research assessor, measurement consistency could not be controlled to the same extent as in a prospective protocol. Because only 62 of 148 screened patients were included in the final analytic cohort, selection bias related to the availability of laboratory, ultrasonographic, and follow-up data may have influenced both the estimated associations and their generalizability. The results should not be interpreted as indicating that serum 25(OH)D or other baseline variables directly affected outcomes after ESWT, as the retrospective observational design limits causal inference. Because the study was conducted at a single center, applicability to other patient populations and practice settings may be limited. The modest sample size increases the possibility of model instability and means that the study was powered only to detect moderate-to-large regression effects. The vitamin D threshold, three-category 25(OH)D, and sex-specific analyses were therefore exploratory and should not be interpreted as definitive subgroup analyses. No formal multiplicity adjustment was applied, and the threshold analyses should therefore be interpreted as supportive rather than confirmatory. A separate pain-specific measure, such as a visual analog scale or numeric rating scale, was not consistently available; therefore, pain intensity could not be evaluated as an independent outcome or covariate. Vitamin D supplementation status, season of blood sampling, dietary intake, sunlight exposure, sex hormone levels including estrogen or estradiol, menopausal status, free or bioavailable 25(OH)D, direct body composition measurements, prior noninvasive treatment exposure, occupational loading, sports-related loading, and adherence to self-management instructions were not consistently available in the medical record and therefore could not be fully controlled. Other ESWT device-adjustable parameters, such as pulse frequency and coupling pressure, were not consistently documented. Additional treatments or self-management measures received outside the institution may also have been incompletely captured. Finally, CET thickness was used as a practical sonographic structural marker, but tendon thickness alone may not capture the full spectrum of ultrasonographic abnormalities in tendinopathy.

Overall, the current study showed that worse 12-week patient-reported function was associated with lower baseline grip strength ratio, higher baseline QuickDASH score, lower serum 25(OH)D level, and greater CET thickness in this retrospective cohort of ESWT-treated patients. Exploratory threshold analysis was consistent with a possible association between 25(OH)D <20 ng/mL and higher 12-week QuickDASH after adjustment. In contrast, baseline grip strength ratio and baseline QuickDASH score showed clearer associations with 12-week grip strength ratio than serum 25(OH)D or CET thickness. Larger prospective studies are needed to assess whether these associations are reproducible and whether vitamin D status adds clinically useful information beyond baseline functional status.

## 5. Conclusions

In this retrospective cohort of patients with lateral epicondylitis treated with outpatient ESWT, lower serum 25(OH)D, interpreted as a vitamin D nutritional-status marker rather than as a direct measure of dietary intake or a nutrition-specific causal exposure, was associated with worse 12-week patient-reported function after adjustment for baseline functional status and other covariates. Serum 25(OH)D was not clearly associated with 12-week grip strength ratio. The <20 ng/mL threshold analysis was exploratory and should not be interpreted as a powered subgroup finding. These findings are best interpreted as observational and hypothesis-generating.

## Figures and Tables

**Table 1:** Baseline Characteristics and Serum 25(OH)D Status of the Study Cohort (*N* = 62).

Variable	Overall (*N* = 62)
Age, years	47.2 ± 16.2
Sex, female	33 (53.2%)
Sex, male	29 (46.8%)
BMI, kg/m^2^	24.6 ± 3.6
Affected side, right	30 (48.4%)
Affected side, left	32 (51.6%)
Dominant arm involvement, yes	45 (72.6%)
Dominant arm involvement, no	17 (27.4%)
Symptom duration, months	9.2 ± 5.4
Grip strength, affected side, kg	16.5 ± 6.2
Grip strength, unaffected side, kg	28.3 ± 7.6
Grip strength ratio	0.58 ± 0.14
QuickDASH at baseline	42.0 ± 17.4
Serum 25(OH)D, ng/mL	21.0 ± 8.4
Serum 25(OH)D status, <20 ng/mL	30 (48.4%)
Serum 25(OH)D status, 20–29.9 ng/mL	22 (35.5%)
Serum 25(OH)D status, ≥30 ng/mL	10 (16.1%)
CET thickness, mm	5.5 ± 1.2

Note. Continuous variables are presented as mean ± standard deviation, and categorical variables as number (percentage). Serum 25(OH)D status categories are presented for descriptive purposes. Abbreviations: BMI, body mass index; CET, common extensor tendon; 25(OH)D, 25-hydroxyvitamin D; QuickDASH, Quick Disabilities of the Arm, Shoulder and Hand.

**Table 2:** Outcomes at Baseline and 12 Weeks.

Outcome Measure	Value
QuickDASH at baseline	42.0 ± 17.4
QuickDASH at 12 weeks	27.0 ± 13.7
Change in QuickDASH (12 weeks—baseline)	−15.0 ± 18.6
Grip strength ratio at baseline	0.58 ± 0.14
Grip strength ratio at 12 weeks	0.76 ± 0.14
Change in grip strength ratio (12 weeks—baseline)	0.17 ± 0.11

Note. Values are presented as mean ± standard deviation. Change values were calculated as 12-week minus baseline. Abbreviation: QuickDASH, Quick Disabilities of the Arm, Shoulder and Hand.

**Table 3:** Multivariable Linear Regression for QuickDASH at 12 Weeks with Rescaled Baseline Predictors.

Predictor	β	95% CI	*p* Value
Grip strength ratio at baseline, per 0.1 increase	−3.91	−5.88 to −1.95	<0.001
QuickDASH at baseline, per 10-point increase	3.80	2.19 to 5.41	<0.001
Serum 25(OH)D, per 10 ng/mL increase	−4.04	−7.17 to −0.91	0.012
Common extensor tendon thickness, per 1 mm increase	5.25	3.05 to 7.46	<0.001
Age, per 1-year increase	0.04	−0.13 to 0.22	0.613
Male sex (vs. female)	2.67	−2.75 to 8.09	0.328
BMI, per 1 kg/m^2^ increase	−0.02	−0.79 to 0.74	0.948

Note. Model R^2^ = 0.527 and adjusted R^2^ = 0.465. Reference category for sex = female. β values are unstandardized coefficients. Coefficients are presented per unit shown in the predictor column. Abbreviations: BMI, body mass index; CET, common extensor tendon; CI, confidence interval; 25(OH)D, 25-hydroxyvitamin D; QuickDASH, Quick Disabilities of the Arm, Shoulder and Hand.

**Table 4:** Multivariable Linear Regression for Grip Strength Ratio at 12 Weeks with Rescaled Baseline Predictors.

Predictor	β	95% CI	*p* Value
Grip strength ratio at baseline, per 0.1 increase	0.070	0.052 to 0.087	<0.001
QuickDASH at baseline, per 10-point increase	−0.019	−0.033 to −0.004	0.011
Serum 25(OH)D, per 10 ng/mL increase	0.023	−0.005 to 0.050	0.110
Common extensor tendon thickness, per 1 mm increase	−0.019	−0.038 to 0.001	0.060
Age, per 1-year increase	−0.0005	−0.0020 to 0.0011	0.558
Male sex (vs. female)	0.027	−0.022 to 0.075	0.274
BMI, per 1 kg/m^2^ increase	0.005	−0.002 to 0.012	0.149

Note. Model R^2^ = 0.618 and adjusted R^2^ = 0.568. Reference category for sex = female. β values are unstandardized coefficients. Coefficients are presented per unit shown in the predictor column. Abbreviations: BMI, body mass index; CET, common extensor tendon; CI, confidence interval; 25(OH)D, 25-hydroxyvitamin D; QuickDASH, Quick Disabilities of the Arm, Shoulder and Hand.

**Table 5:** Exploratory Threshold Analysis Using Serum 25(OH)D Deficiency Defined as <20 ng/mL.

Outcome	Threshold Contrast	β	95% CI	*p* Value	Adjusted R^2^
QuickDASH at 12 weeks	25(OH)D <20 vs. ≥20 ng/mL	6.43	1.17 to 11.69	0.017	0.459
Grip strength ratio at 12 weeks	25(OH)D <20 vs. ≥20 ng/mL	−0.008	−0.056 to 0.039	0.723	0.548

Note. Each model was adjusted for baseline grip strength ratio, baseline QuickDASH score, CET thickness, age, sex, and BMI. Reference category for serum 25(OH)D status = ≥20 ng/mL. β values are unstandardized coefficients. Abbreviations: BMI, body mass index; CET, common extensor tendon; CI, confidence interval; 25(OH)D, 25-hydroxyvitamin D; QuickDASH, Quick Disabilities of the Arm, Shoulder and Hand.

**Table 6:** Exploratory Analysis According to Three-Category Serum 25(OH)D Status.

Outcome	25(OH)D Status Contrast	β	95% CI	*p* Value	Overall Status *p* Value	Adjusted R^2^
QuickDASH at 12 weeks	<20 vs. ≥30 ng/mL	8.29	0.81 to 15.77	0.030	0.048	0.454
QuickDASH at 12 weeks	20–29.9 vs. ≥30 ng/mL	2.77	−5.10 to 10.63	0.483		
Grip strength ratio at 12 weeks	<20 vs. ≥30 ng/mL	−0.053	−0.119 to 0.012	0.109	0.153	0.570
Grip strength ratio at 12 weeks	20–29.9 vs. ≥30 ng/mL	−0.067	−0.136 to 0.002	0.058		

Note. Models were adjusted for baseline grip strength ratio, baseline QuickDASH score, CET thickness, age, sex, and BMI. The reference category for serum 25(OH)D status was ≥30 ng/mL. The ≥30 ng/mL group included 10 patients, so this analysis should be considered exploratory. β values are unstandardized coefficients. Abbreviations: BMI, body mass index; CET, common extensor tendon; CI, confidence interval; 25(OH)D, 25-hydroxyvitamin D; QuickDASH, Quick Disabilities of the Arm, Shoulder and Hand. No formal multiplicity adjustment was applied.

## Data Availability

De-identified individual-level data supporting the conclusions of this article are not publicly available because of institutional and ethical restrictions related to patient privacy. Reasonable requests for access to de-identified data may be considered by the corresponding author, subject to approval by the Institutional Review Board of Kyung Hee University Hospital at Gangdong and applicable institutional data-protection requirements.

## Supplementary Materials

The following supporting information can be downloaded at https://www.mdpi.com/article/10.3390/nu18132152/s1: Table S1: Sensitivity analysis after additional adjustment for symptom duration; Table S2: Sensitivity analysis after additional adjustment for dominant arm involvement; Table S3: Sex-stratified baseline characteristics and outcomes; Table S4: Exploratory sex-stratified and sex-interaction analyses; Table S5: Regression diagnostics and bootstrap stability analyses.

## Abbreviations

ANCOVA	Analysis of covariance
BMI	Body mass index
CET	Common extensor tendon
CI	Confidence interval
ESWT	Extracorporeal shock wave therapy
QuickDASH	Quick Disabilities of the Arm, Shoulder and Hand
