# Supplementary material for: Serum 25-Hydroxyvitamin D Status and 12-Week Functional Outcomes After Extracorporeal Shock Wave Therapy for Lateral Epicondylitis: A Retrospective Cohort Study
Source: Nutrients. 2026 Jul 2;18(13):2152. doi: 10.3390/nu18132152 (PMC13363471; doi:10.3390/nu18132152)
Supplement: Supplementary file 1 [file nutrients-18-02152-s001.zip › nutrients-4363181-supplementary.pdf]

Supplementary Table S1. Sensitivity Analysis After Additional Adjustment for Symptom Duration.

Outcome	Predictor	β	95% CI	p value	Adjusted R^2
QuickDASH at 12 weeks	Grip strength ratio at baseline, per 0.1 increase	-3.92	-5.91 to -1.93	<0.001	0.455
	QuickDASH at baseline, per 10-point increase	3.76	2.07 to 5.46	<0.001	
	Serum 25(OH)D, per 10 ng/mL increase	-4.09	-7.31 to -0.86	0.014	
	Common extensor tendon thickness, per 1 mm increase	5.28	3.03 to 7.53	<0.001	
	Age, per 1-year increase	0.05	-0.13 to 0.23	0.602	
	Male sex (vs female)	2.73	-2.80 to 8.26	0.327	
	BMI, per 1 kg/m² increase	-0.03	-0.80 to 0.74	0.945	
	Symptom duration, per 1-month increase	-0.04	-0.59 to 0.51	0.883	
Grip strength ratio at 12 weeks	Grip strength ratio at baseline, per 0.1 increase	0.070	0.052 to 0.087	<0.001	0.562
	QuickDASH at baseline, per 10-point increase	-0.020	-0.035 to -0.005	0.011	
	Serum 25(OH)D, per 10 ng/mL increase	0.021	-0.007 to 0.050	0.144	
	Common extensor tendon thickness, per 1 mm increase	-0.018	-0.038 to 0.002	0.076	
	Age, per 1-year increase	-0.0004	-0.0020 to 0.0013	0.657	
	Male sex (vs female)	0.028	-0.021 to 0.077	0.253	
	BMI, per 1 kg/m² increase	0.005	-0.002 to 0.012	0.157	
	Symptom duration, per 1-month increase	-0.001	-0.006 to 0.004	0.630	

Note. Models were additionally adjusted for symptom duration in months. Reference category for sex = female. β values are unstandardized coefficients. Coefficients are presented per unit shown in the predictor column. Abbreviations: BMI, body mass index; CET, common extensor tendon; CI, confidence interval; 25(OH)D, 25-hydroxyvitamin D; QuickDASH, Quick Disabilities of the Arm, Shoulder and Hand.

Supplementary Table S2. Sensitivity Analysis After Additional Adjustment for Dominant Arm Involvement.

Outcome	Predictor	β	95% CI	p value	Adjusted R²
QuickDASH at 12 weeks	Grip strength ratio at baseline, per 0.1 increase	-3.95	-5.94 to -1.96	<0.001	0.457
	QuickDASH at baseline, per 10-point increase	3.86	2.22 to 5.51	<0.001	
	Serum 25(OH)D, per 10 ng/mL increase	-4.14	-7.33 to -0.95	0.012	
	Common extensor tendon thickness, per 1 mm increase	5.18	2.93 to 7.42	<0.001	
	Age, per 1-year increase	0.04	-0.14 to 0.22	0.657	
	Male sex (vs female)	2.70	-2.76 to 8.16	0.326	
	BMI, per 1 kg/m² increase	-0.05	-0.83 to 0.73	0.895	
	Dominant arm involvement, yes (vs no)	-1.41	-7.52 to 4.70	0.646	
Grip strength ratio at 12 weeks	Grip strength ratio at baseline, per 0.1 increase	0.070	0.052 to 0.087	<0.001	0.560
	QuickDASH at baseline, per 10-point increase	-0.019	-0.033 to -0.004	0.013	
	Serum 25(OH)D, per 10 ng/mL increase	0.023	-0.006 to 0.051	0.116	
	Common extensor tendon thickness, per 1 mm increase	-0.019	-0.039 to 0.001	0.066	
	Age, per 1-year increase	-0.0005	-0.0020 to 0.0011	0.568	
	Male sex (vs female)	0.026	-0.022 to 0.075	0.279	
	BMI, per 1 kg/m² increase	0.005	-0.002 to 0.012	0.155	
	Dominant arm involvement, yes (vs no)	0.001	-0.053 to 0.056	0.963	

Note. Models were additionally adjusted for dominant arm involvement. Reference category for sex = female. Reference category for dominant arm involvement = no. β values are unstandardized coefficients. Coefficients are presented per unit shown in the predictor column. Abbreviations: BMI, body mass index; CET, common extensor tendon; CI, confidence interval; 25(OH)D, 25-hydroxyvitamin D; QuickDASH, Quick Disabilities of the Arm, Shoulder and Hand.

Supplementary Table S3. Sex-stratified baseline characteristics and outcomes.

Variable	Female (N = 33)	Male (N = 29)	p value
Age, years	48.9 ± 17.0	45.4 ± 15.2	0.396
BMI, kg/m²	24.4 ± 3.7	24.7 ± 3.5	0.811
Symptom duration, months	8.3 ± 5.4	10.2 ± 5.2	0.151
Grip strength, affected side, kg	17.9 ± 7.1	14.9 ± 4.5	0.053
Grip strength, unaffected side, kg	28.7 ± 7.8	28.0 ± 7.4	0.723
Grip strength ratio at baseline	0.61 ± 0.13	0.55 ± 0.15	0.067
QuickDASH at baseline	45.3 ± 19.0	38.2 ± 14.7	0.103
Serum 25(OH)D, ng/mL	20.6 ± 8.6	21.5 ± 8.2	0.692
CET thickness, mm	5.4 ± 1.1	5.6 ± 1.3	0.386
QuickDASH at 12 weeks	25.4 ± 12.3	28.8 ± 15.2	0.332
Grip strength ratio at 12 weeks	0.76 ± 0.12	0.75 ± 0.15	0.856
Change in QuickDASH	-19.9 ± 18.5	-9.3 ± 17.3	0.023
Change in grip strength ratio	0.15 ± 0.11	0.21 ± 0.10	0.030
Affected side, right	16 (48.5%)	14 (48.3%)	0.987
Dominant arm involvement, yes	24 (72.7%)	21 (72.4%)	0.978
Serum 25(OH)D status, <20 ng/mL	17 (51.5%)	13 (44.8%)	0.871
Serum 25(OH)D status, 20-29.9 ng/mL	11 (33.3%)	11 (37.9%)	
Serum 25(OH)D status, ≥30 ng/mL	5 (15.2%)	5 (17.2%)	
QuickDASH responder, ≥12-point improvement	22 (66.7%)	13 (44.8%)	0.084

Note. Continuous variables are presented as mean ± standard deviation, and categorical variables as number (percentage). p values were calculated using Welch t-tests for continuous variables and chi-square or Fisher exact tests for categorical variables, as appropriate. QuickDASH responder was defined as a ≥12-point improvement from baseline to 12 weeks. Abbreviations: BMI, body mass index; CET, common extensor tendon; 25(OH)D, 25-hydroxyvitamin D; QuickDASH, Quick Disabilities of the Arm, Shoulder and Hand.

Supplementary Table S4. Exploratory sex-stratified and sex-interaction analyses.

Analysis	Outcome	β	95% CI	p value
Female subgroup: baseline grip strength ratio, per 0.1 increase	12-week QuickDASH	-4.27	-6.78 to -1.76	0.002
Male subgroup: baseline grip strength ratio, per 0.1 increase	12-week QuickDASH	-3.54	-7.72 to 0.64	0.093
Female subgroup: baseline grip strength ratio, per 0.1 increase	12-week grip strength ratio	0.064	0.039 to 0.090	<0.001
Male subgroup: baseline grip strength ratio, per 0.1 increase	12-week grip strength ratio	0.067	0.034 to 0.100	<0.001
Sex × baseline grip strength ratio interaction	12-week QuickDASH	0.94	-3.13 to 5.00	0.646
Sex × baseline grip strength ratio interaction	12-week grip strength ratio	0.009	-0.027 to 0.045	0.620

Note. β values are unstandardized coefficients. Sex-stratified models were adjusted for baseline QuickDASH score, serum 25(OH)D level, common extensor tendon thickness, age, and body mass index. Interaction models included sex, baseline grip strength ratio, the sex-by-baseline grip strength ratio interaction term, baseline QuickDASH score, serum 25(OH)D level, common extensor tendon thickness, age, and body mass index. Baseline grip strength ratio coefficients are presented per 0.1 increase. Abbreviations: CI, confidence interval; 25(OH)D, 25-hydroxyvitamin D; QuickDASH, Quick Disabilities of the Arm, Shoulder and Hand.

Supplementary Table S5. Regression diagnostics and bootstrap stability analyses.

**Diagnostic / stability item**	QuickDASH outcome model	Grip strength ratio outcome model
Maximum variance inflation factor	1.19	1.19
Breusch-Pagan test	p = 0.258	p = 0.805
Bootstrap 95% CI for serum 25(OH)D coefficient	-7.25 to -1.24 per 10 ng/mL	-0.007 to 0.050 per 10 ng/mL
Leave-one-out refitting	Main associations retained direction	Main associations retained direction

Note. Diagnostic analyses were performed for the QuickDASH and grip strength ratio outcome models. Bootstrap intervals were obtained from 5,000 non-parametric resamples. Abbreviations: CI, confidence interval; 25(OH)D, 25-hydroxyvitamin D; QuickDASH, Quick Disabilities of the Arm, Shoulder and Hand.
